# The crystal structure of *Vibrio cholerae* (6-4) photolyase reveals interactions with cofactors and a DNA-binding region

**DOI:** 10.1016/j.jbc.2022.102794

**Published:** 2022-12-14

**Authors:** Baris Cakilkaya, Ibrahim Halil Kavakli, Hasan DeMirci

**Affiliations:** 1 Department of Molecular Biology and Genetics, Koc University, Istanbul, Turkey; 2 Department Chemical and Biological Engineering, Koc University, Istanbul, Turkey; 3 Koc University Isbank Center for Infectious Diseases (KUIS-CID), Koc University, Istanbul, Turkey; 4 PULSE Institute, SLAC National Accelerator Laboratory, Menlo Park, California, USA

**Keywords:** photolyase, (6-4) lesioned photoproduct, X-ray crystallography, flavin adenine dinucleotide, 6,7-dimethyl 8-ribityl-lumazin, CPD, cyclobutane pyrimidine dimer, CRY, cryptochrome, DASH, Drosophila, Arabidopsis, Synechocystis, and Human, DMRL, 6,7-dimethyl 8-ribityl-lumazin, FAD, flavin adenine dinucleotide, PL, photolyase, PDB, Protein Data Bank

## Abstract

Photolyases (PLs) reverse UV-induced DNA damage using blue light as an energy source. Of these PLs, (6-4) PLs repair (6-4)-lesioned photoproducts. We recently identified a gene from *Vibrio cholerae* (*Vc*) encoding a (6-4) PL, but structural characterization is needed to elucidate specific interactions with the chromophore cofactors. Here, we determined the crystal structure of *Vc* (6-4) PL at 2.5 Å resolution. Our high-resolution structure revealed that the two well-known cofactors, flavin adenine dinucleotide and the photoantenna 6,7-dimethyl 8-ribityl-lumazin (DMRL), stably interact with an α-helical and an α/β domain, respectively. Additionally, the structure has a third cofactor with distinct electron clouds corresponding to a [4Fe-4S] cluster. Moreover, we identified that Asp106 makes a hydrogen bond with water and DMRL, which indicates further stabilization of the photoantenna DMRL within *Vc* (6-4) PL. Further analysis of the *Vc* (6-4) PL structure revealed a possible region responsible for DNA binding. The region located between residues 478 to 484 may bind the lesioned DNA, with Arg483 potentially forming a salt bridge with DNA to stabilize further the interaction of *Vc* (6-4) PL with its substrate. Our comparative analysis revealed that the DNA lesion could not bind to the *Vc* (6-4) PL in a similar fashion to the *Drosophila melanogaster* (*Dm*, (6-4)) PL without a significant conformational change of the protein. The 23rd helix of the bacterial (6-4) PLs seems to have remarkable plasticity, and conformational changes facilitate DNA binding. In conclusion, our structure provides further insight into DNA repair by a (6-4) PL containing three cofactors.

The cryptochrome (CRY)/photolyase (PL) family is a large family of flavoproteins that absorb near UV–visible light (300–500 nm range) and has diverse functions depending on the type of organism (1, 2). CRYs are the circadian photoreceptors in *Drosophila* and plants, while they act as transcriptional repressors of the circadian clock in mammals (2, 3, 4). PLs are enzymes that repair UV-induced DNA damage (5, 6, 7). Although phylogenetic analyses revealed different classes of PLs, there are three different types of PLs based on their repair activities: Cyclobutane Pyr dimer (CPD) PLs, (6–4) PLs, and *Drosophila*, *Arabidopsis*, *Synechocystis*, and Human (DASH)-type CRY (CRY-DASH) (8, 9, 10). CPD PLs repair mainly CPDs (Pyr<>Pyr), while (6–4) PLs repair Pyr-Pyr photoproducts (Pyr [6–4] Pyr) using blue light (350–500 nm) as the energy source (1, 6). CRY-DASHs have quite diverse physiological functions including ssDNA PL activity (11). PLs are monomeric proteins with molecular masses of 50 to 61 kDa and possess two chromophores (12). Flavin adenine dinucleotide (FAD) is an essential cofactor for the catalytic activity of PLs and acts as a catalytic cofactor in all PLs (5, 12). The second chromophore, which acts as a photoantenna, varies depending on the organism. The following photoantenna are being found: methenyltetrahydrofolate, 8-hydroxy-7,8-didemethyl-5-deazariboflavin, FAD, FMN, or 6,7-dimethyl 8-ribityl-lumazin (DMRL) depending on the type of PL (13, 14). The (6–4) PLs had previously been thought to exist only in eukaryotes. However, studies with different bacteria revealed the presence of (6–4) PLs (15, 16, 17). These bacterial (6–4) PLs are distinct in that they contain [4 Fe-4 S] as well as DMRL and FAD cofactors (15, 17).

Crystal structures of various PLs have been determined by X-ray crystallography. The first available crystal structure is *Escherichia coli* CPD PL (18). Subsequent structures have been obtained from different organismal PLs including *Anacystis nidulans* (19), *Thermus thermophilus* (20), (6–4) PLs of *Arabidopsis thaliana* (21) and *Agrobacterium tumefaciens* (13). The comparison of the crystal structures of various homologous PLs revealed valuable information about their structural features and enzyme mechanisms. PLs are made up of two well-defined domains which are α/β and α-helical domains. The α-helical domain is associated with FAD, while α/β domain is the binding site for the second chromophore (12). These two domains are connected by a long flexible loop.

*Vibrio cholerae* O1 bivar Tor str. N16961 has a CPD PL that repairs T<>T dimers and two CRY-DASHs that repair T<>T dimers in ssDNA (11, 22). Our previous study with a gene (*Vca0809*) in *V. cholerae* highly expressed upon exposure of the organism to blue light (23). Our further characterization of this gene revealed that it encodes (6-4) PL with FAD and DMRL and consists of the [4Fe-4S] domain (15). Here, we determined the crystal structure of *Vc* (6-4) PL to 2.5 Å resolution. Our crystal structure comparison revealed a putative DNA-binding region of *Vc* (6-4) PL.

## Results

### Crystal structure of *Vc* (6-4) PL

**Figure 1:**
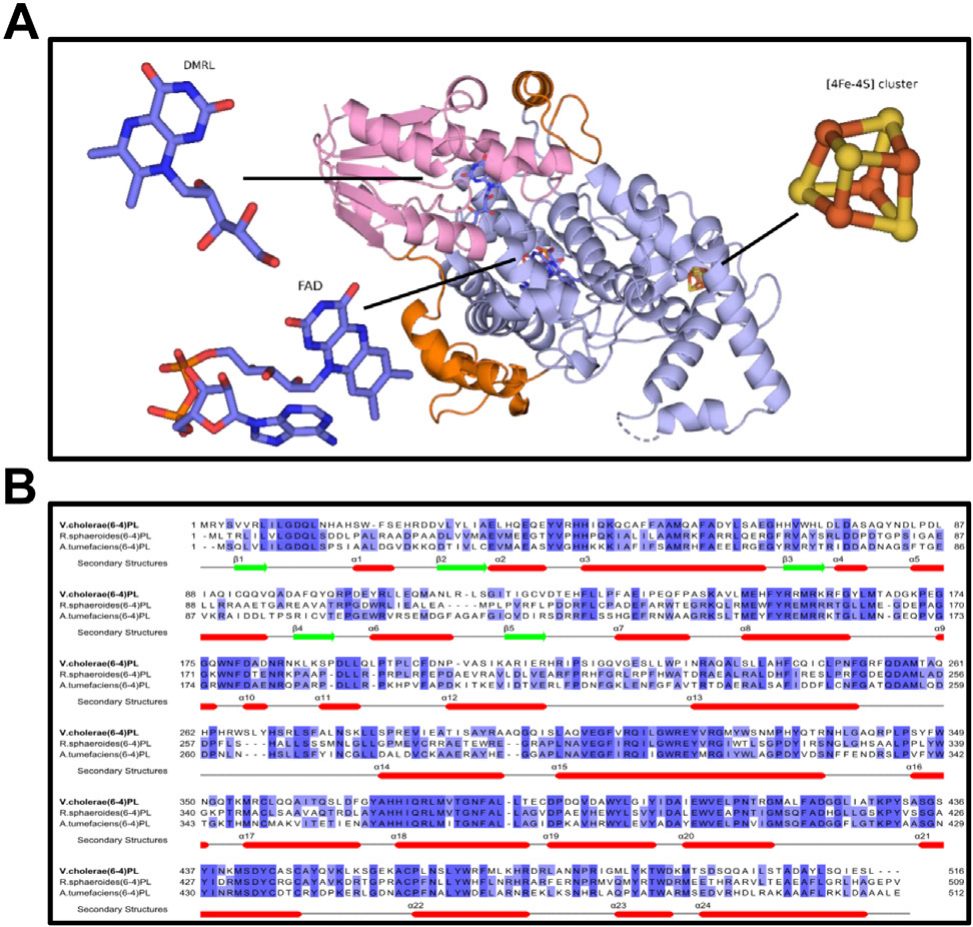
**Crystal structure and sequence comparison of *Vc* (6-4) PL.***A*, overview of *Vc* (6-4) photolyase (PL) structure, *pink* is α-/β-domain, *light blue* is catalytic domain, and *orange* is the interdomain linker region. Cofactor places are indicated by the *black line*. *B*, sequence alignment of bacterial (6-4) PLs with available crystal structures and secondary structures of *Vc* (6-4) PL are shown. *Red lines* are α helixes, and *green lines* are β sheets. Percentage identity coloring is used in JALVIEW software. DMRL, 6,7-dimethyl 8-ribityl-lumazin; FAD, flavin adenine dinucleotide.

We recently identified a (6-4) PL from *V. cholerae* O1 bivar Tor str. N16961(*Vc*) which possesses the catalytic cofactor FAD, an antenna chromophore DMRL, and an additional cofactor [4Fe-4S] cluster (15). We determined the crystal structure of *Vc* (6-4) PL at 2.5 Å resolution at cryogenic temperature at the Turkish Light Source known as “*Turkish DeLight*” (24, 25). The structure contains all the cofactors with well-defined electron densities and contains a total of 24 α helixes and five β sheets (Fig. 1*A*). It consists of two domains: an α-helical domain that is associated with FAD and α/β domain which interacts with DMRL cofactor. The sequence alignment of *Vc* (6-4) PL with *A. tumefaciens* (*At*) and *Rhodobacter sphaeroides* (*Rs*) (6-4) PLs showed that it has 38.4% and 43.1% sequence similarity, respectively (Fig. 1*B*).

### DMRL-binding domain

**Figure 2:**
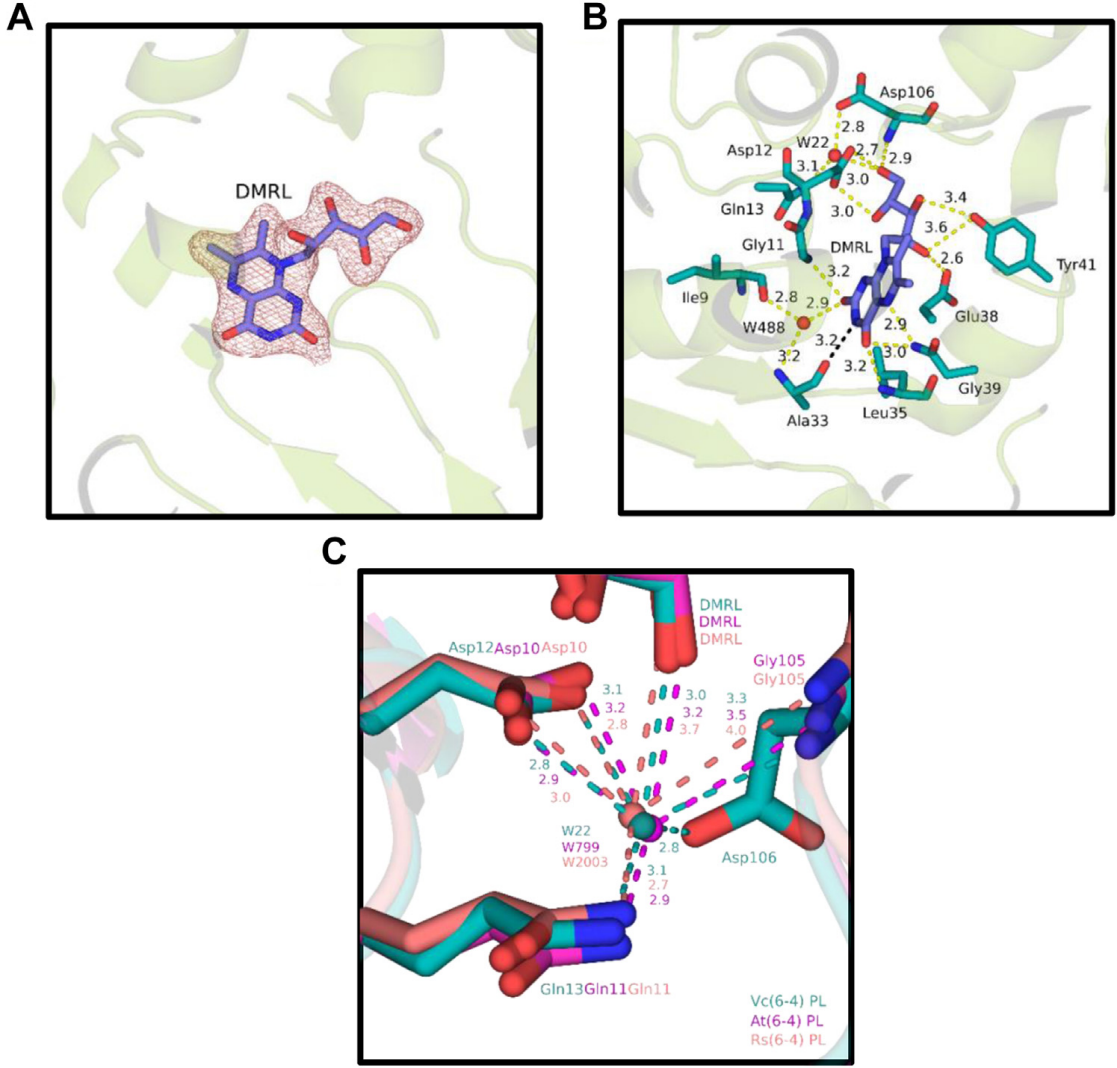
**DMRL interactions and comparisons with other (6-4) photolyases.** Every distance is in units of Armstrong (Å). *A*, electron cloud of DMRL at σA-weighted 2Fo-Fc electron density map contoured at 1.0σ in deep salmon mesh and *Vc* (6-4) PL cartoon in *split pea color*. *B*, possible interaction partners of DMRL. *Yellow dashes* represent hydrogen bondings, *black dashes* indicate Van der Waals interactions, and *red dots* are water molecules. *C*, additional water molecules that might stabilize the DMRL interaction. *Deep teal*, *magenta*, and *deep salmon colors* indicate *Vc* (6-4) PL, *At* (6-4) PL (PDB ID: 4DJA), and *Rs* (6-4) PL (PDB ID: 3ZXS), respectively. Their superimposed structures have RMSD: 0.834 Å (*At* (6-4) PL) and RMSD: 0.876 Å (*Rs* (6-4) PL). Each *dashed line* represents hydrogen bonding of species with corresponding color. DMRL, 6,7-dimethyl 8-ribityl-lumazin; PDB, Protein Data Bank; PL, photolyase.

DMRL is a photoantenna chromophore that absorbs light more efficiently than FAD for increasing catalytic efficiency (13). In our crystal structure, there is a very well-defined electron density of the DMRL and interacting amino acid residues (Fig. 2*A*). The aromatic ring of DMRL interacts with nitrogen atoms of Gln11, Leu35, Gln39, and oxygen atom of Ala33 (Fig. 2*B*). Additionally, the aromatic ring of DMRL interacts with Trp488, Ala33, and Ile9. The DMRL's ribityl group forms hydrogen bonds with the oxygen atoms of Glu38, Tyr41, Asp12, and Asp10's amino group. When we compare the interaction partners of DMRL in *Vc* (6-4) PL with other known bacterial (6-4) PLs, almost all the interactions are well conserved with a few exceptions. First, the Cys32 carboxyl group of *At* (6-4) PL interacts with the aromatic ring in DMRL (Protein Data Bank (PDB) ID: 4DJA), while in both *Vc* and *Rs* (6-4) PLs, DMRL interacts with the main chain carbonyl group of Ala33. We identified a critical water molecule (W22) that interacts with Asp12, Gln13, Asp106, and DMRL in *Vc* (6-4) PL (Fig. 2*C*). Analyses of other bacterial (6-4) PLs have shown similar conserved motifs (Fig. 2*C*). Such motifs increase the overall stability of the DMRL within the enzyme. However, the W22 molecule interacts with Asp106 of the *Vc* (6-4) PL with a distance of 2.8 Å, while the same interaction occurs with Gly105 of other bacterial (6-4) PLs with a distance of over 3.3 Å. These differences might increase the affinity of DMRL with *Vc* (6-4) PL.

### FAD-binding domain

**Figure 3:**
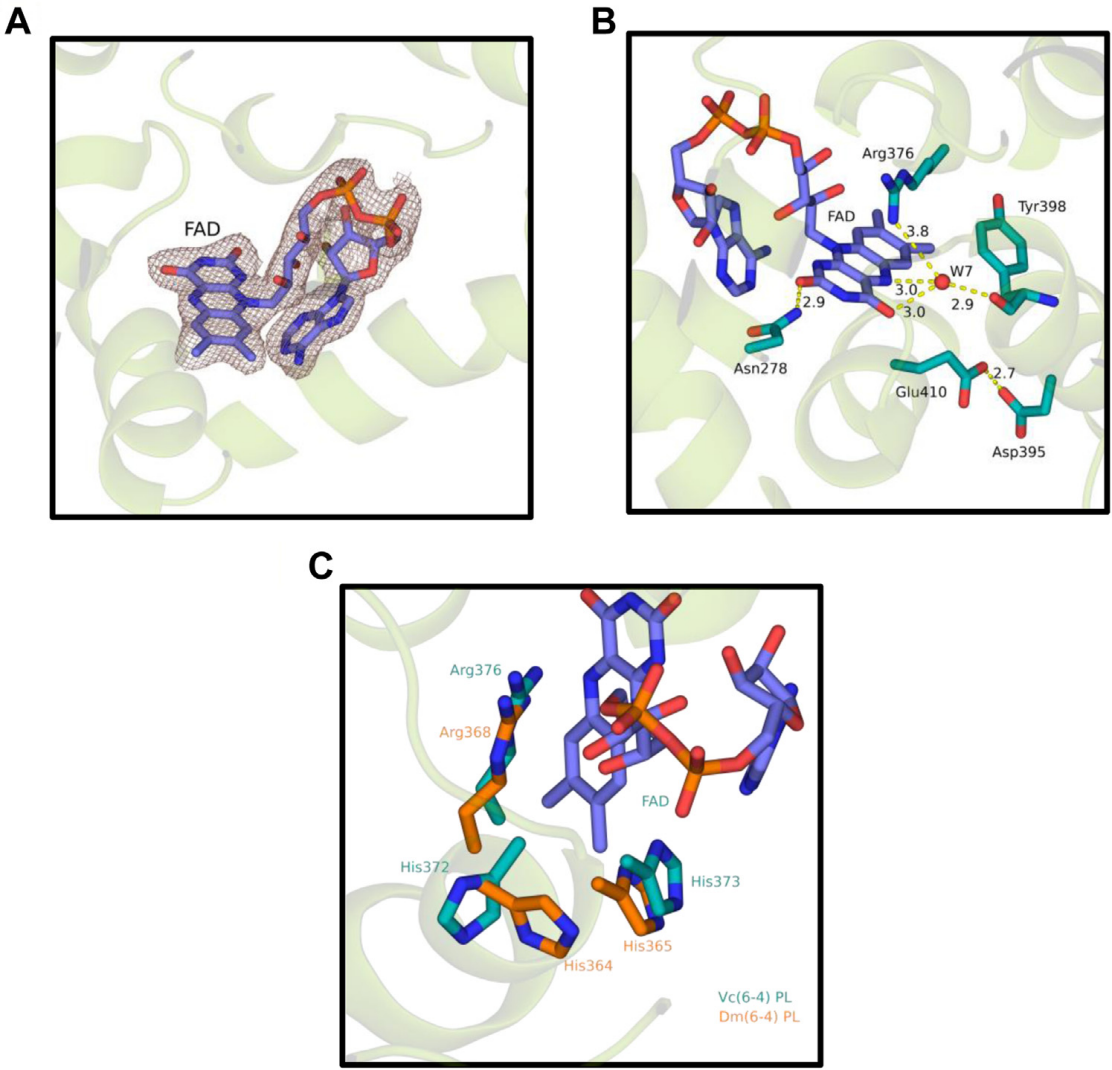
**FAD interactions and comparisons with other (6-4) photolyases.** Every distance is in units of Angstrom (Å). *A*, electron cloud of FAD at σA-weighted 2Fo-Fc electron density map contoured at 1.0σ in deep salmon mesh and *Vc* (6-4) PL cartoon in *split pea color*. *B*, possible interaction partners of FAD. *Yellow dashes* represent hydrogen bondings; *red dots* are water molecules. *C*, superimposed structure of *Vc* (6-4) PL and *Dm* (6-4) PL (PDB ID: 7AYV) at RMSD: 4.350 Å. *Teal color* represents *Vc* (6-4) PL and *tv_orange color* represents *Dm* (6-4) PL. FAD, flavin adenine dinucleotide; PDB, Protein Data Bank; PL, photolyase.

FAD has a U-shaped conformation in the α-helical domain (Fig. 3*A*). The N5 and O4 of FAD form hydrogen bonds with W7 and additional hydrogen bonds with the carbonyl group of Tyr398 and NH1 atom of Arg376 residue (Fig. 3*B*). As previously suggested for other PLs (17) these critical interactions may also serve a stabilization function *Vc* (6-4) PL. Unlike *At* (6-4) PL or *Rs* (6-4) PL, *Vc* (6-4) PL Glu410 amino acid faces away from FAD and interacts with Asp395 rather than a His365 residue (Fig. 3*B*). This results in a much-relaxed interaction between the residues due to hydrogen bonding formation rather than salt bridge formation in *Vc* (6-4) PL.

Although the reaction mechanism of the CPD PLs has been elucidated, (6-4) PLs are not yet fully characterized. There are different mechanisms that are proposed for the (6–4) PL mechanism during DNA repair (7, 14, 26, 27). In fact, mutagenesis studies with *Drosophila melanogaster (Dm* (6-4)) PL indicated that replacement of His365 with Asn365 results in the loss of its DNA repair activity, while mutagenesis of His369 into Met369 results in a highly reduced activity of *Dm* (6-4) PL (28). However, mutagenesis studies with corresponding amino acid residues in *Xenopus* (6-4) PL showed a complete loss of activity (29). Femtosecond spectroscopy and site-directed mutagenesis suggest initial electron transfer from excited flavin induces transfer of a proton from a histidine on the active site of the enzyme to the (6–4) photoproduct (7, 30).

Catalytically active His373 (corresponds to His365 of the *Dm* (6-4) PL) is conserved in *Vc* (6-4) PL (Fig. 3*C*). In addition, there are other conserved amino acid residues in the motif. For instance, His372 and Arg376 (corresponds to His364 and Arg368 of the *Dm* (6-4) PL) are conserved in *Vc* (6-4) PL (Fig. 3*C*). However, the conformation of the *Vc* (6-4) PL His372 is significantly different from the *Dm* (6-4) PL His364, which may suggest an alternate function of this residue.

### [4Fe-4S] cluster

**Figure 4:**
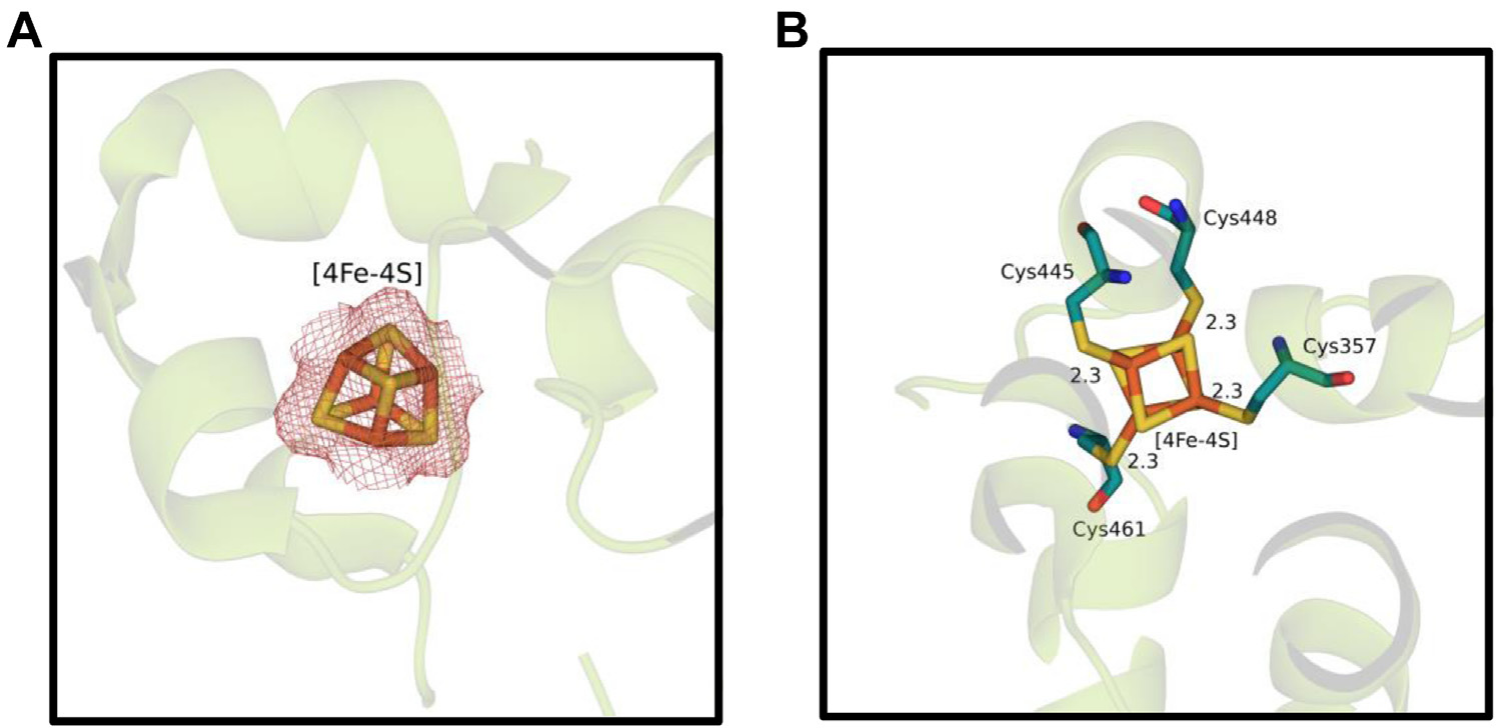
**[4Fe-4S] cluster interactions.***A*, electron cloud of [4Fe-4S] cluster at σA-weighted 2Fo-Fc electron density map contoured at 1.0σ in deep salmon mesh and *Vc* (6–4) PL cartoon in *split pea color*. *B*, direct interactions of [4Fe-4S] clusters with surrounding amino acids of *Vc* (6–4) PL. Every distance is in units of Angstrom (Å). PDB, Protein Data Bank; PL, photolyase.

The high-resolution *Vc* (6-4) PL structure contains an iron–sulfur cluster with an unknown function and a well-defined electron density (Fig. 4*A*). Its interactions with the surrounding cysteine amino acids are summarized in Figure 4*B*. Cys357, Cys445, Cys448, and Cys461 sulfide atoms make bonds with Fe atoms in the cluster with each bond at 2.3 Å distance. The function of the [4Fe-4S] cluster is currently unknown; however, its distance to the FAD is 17 Å, which indicates it does not have a catalytic role in the DNA repair due to its large distance to the active site region.

[4Fe-4S] clusters are well-known for their oxygen sensitivity, and under aerobic conditions, they quickly decompose into [3Fe-4S] clusters and can further dissociate into [2Fe-2S]. Mostly, the decomposition of the cluster deactivates the cluster-containing enzymes, and handling and purification of these enzymes require using a glovebox to prevent decomposition of the oxygen-sensitive [4Fe-4S] clusters. In the case of *Vc* (6-4) PL, the entire expression, purification, and crystallization procedures were performed in atmospheric conditions that took months. In our structure, there was no observable damage in the [4Fe-4S] cluster, which might indicate the cluster in the PL is unusually oxygen tolerant.

### DNA-binding region of *Vc* (6-4) PL

**Figure 5:**
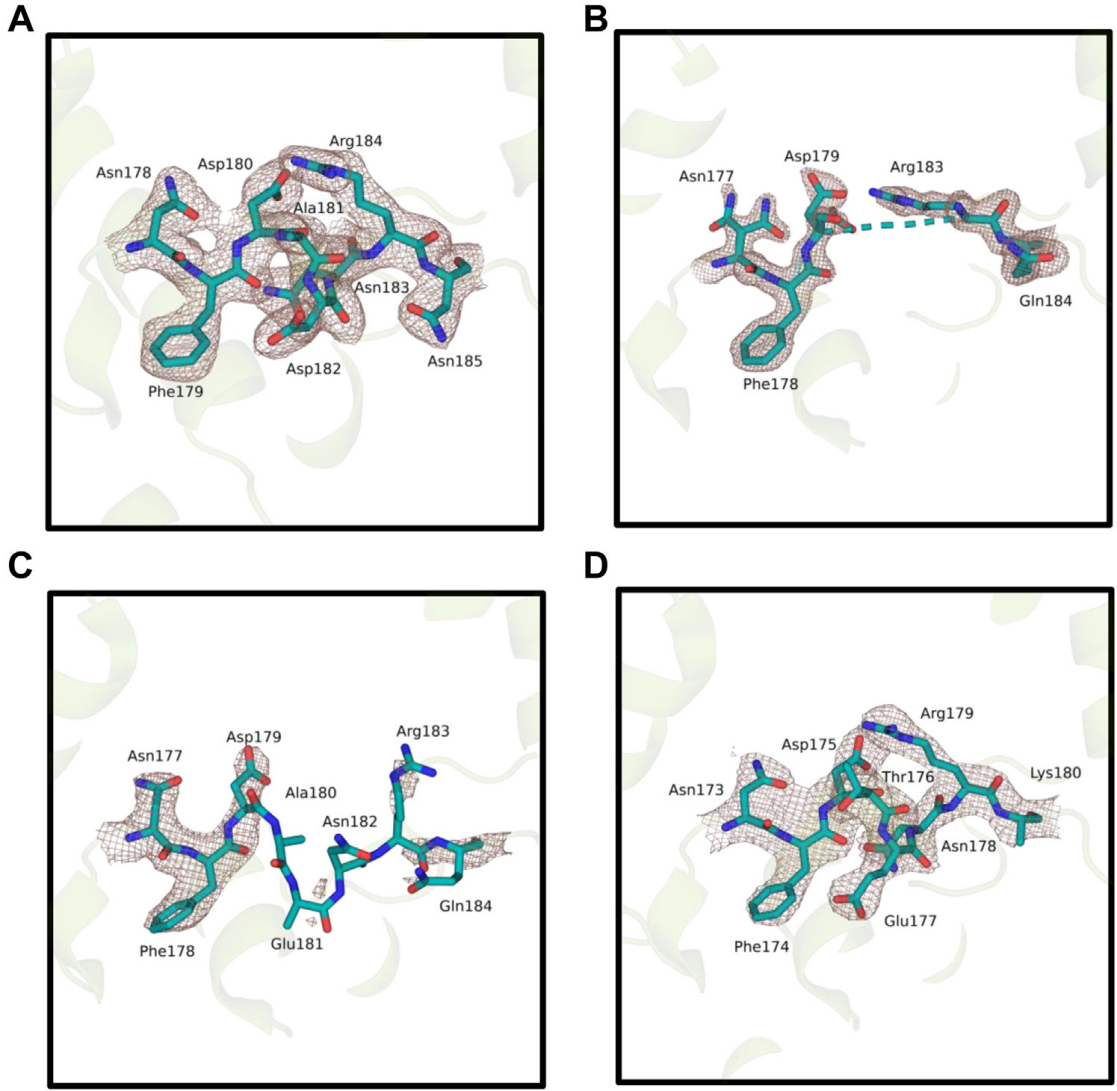
**Possible DNA-binding site comparison with structurally known WT bacterial (6-4) photolyases.** Possible DNA-binding sites are drawn with electron clouds at σA-weighted 2Fo-Fc electron density map contoured at 1.0σ in deep salmon mesh and (6-4) photolyase (PL) cartoon in *split pea color*. *A*, *Vc* (6-4) PL, *B*, *At* (6-4) PL cryogenic (PDB ID: 4DJA), *C*, *At* (6-4) PL ambient (PDB ID: 6DD6), and *D*, *Rs* (6-4) PL (PDB ID: 3ZXS). In (*B*), three amino acids are missing, which are Ala180, Glu181, and Asn182, due to lack of electron clouds. PDB, Protein Data Bank.

The DNA-binding region of the *Vc* (6-4) PL has a defined electron density that covers the amino acids Asn178, Phe179, Asp180, Ala181, Asp182, Asn183, Arg184, and Asn185 (Fig. 5*A*). In contrast, this region has not a well-defined electron density in *At* (6-4) PL structures obtained in either cryogenic (PDB ID: 4DJA) or ambient temperature (PDB ID: 6DD6) conditions (Fig. 5, *B* and *C*). A similar analysis was carried out with *Rs* (6-4) PL, where there is a well-defined electron density in the same region (Fig. 5*D*). These results suggest that while this region is highly stable in *Rs* (6-4) PL and *Vc* (6-4) PL, it is flexible and disordered in *At* (6-4) PL structure and, therefore, this region has species-specific stability differences.

**Figure 6:**
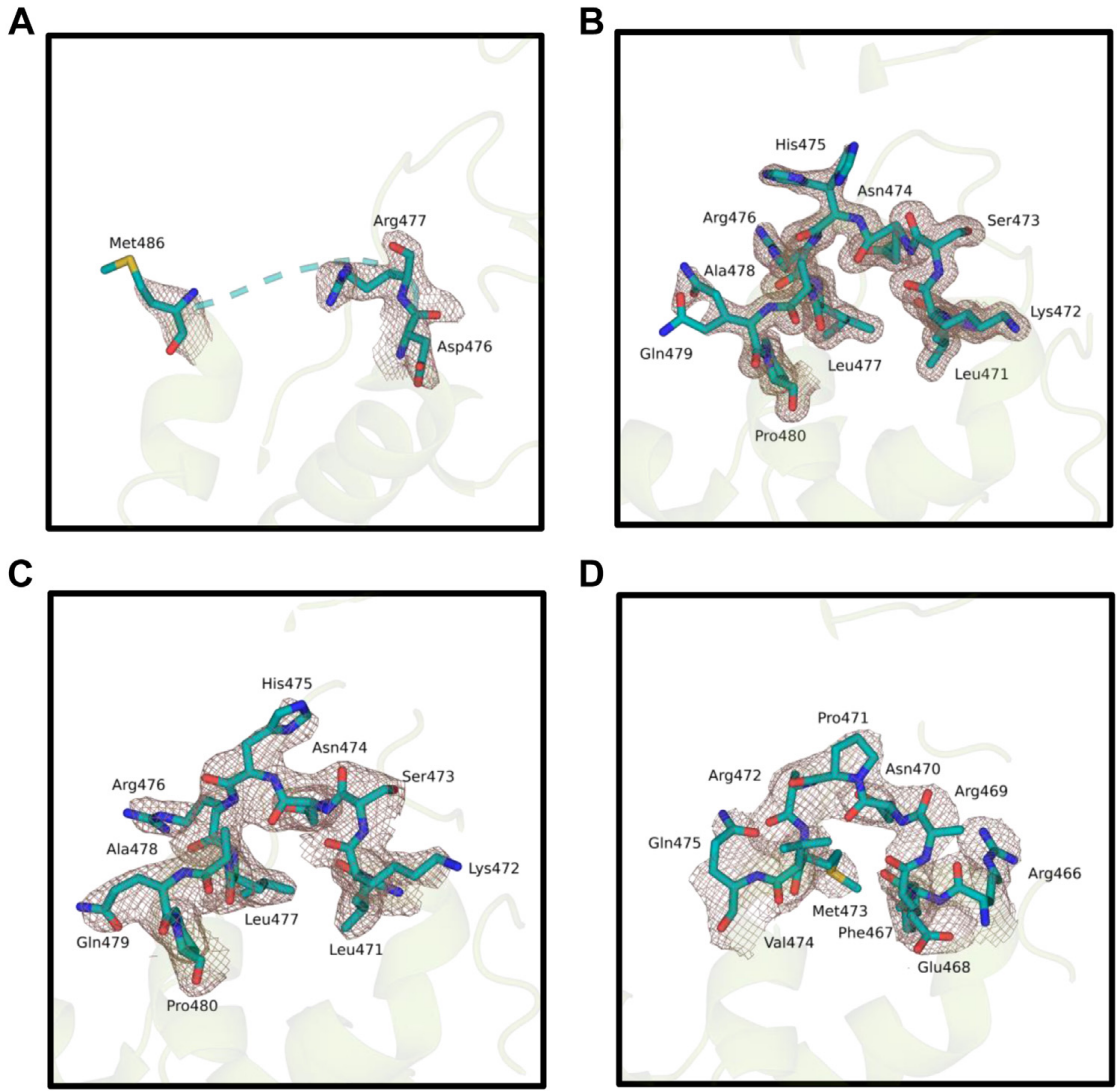
**Possible DNA-binding domain comparison with structurally known WT bacterial (6-4) photolyase.** Each possible DNA-binding site’s electron cloud is drawn at σA-weighted 2Fo-Fc electron density map contoured at 1.0σ in deep salmon mesh and (6-4) photolyase (PL) cartoon in *split pea color*. *A*, *Vc* (6-4) PL, *B*, *At* (6-4) PL cryogenic (PDB ID: 4DJA), *C*, *At* (6-4) PL ambient (PDB ID: 6DD6), and (*D*) *Rs* (6-4) PL (PDB ID: 3ZXS). In (*A*), eight amino acids are missing, which are Leu478, Ala479, Asn480, Asn481, Pro482, Arg483, Ile484, and Gly485 due to lack of electron density cloud. PDB, Protein Data Bank.

**Figure 7:**
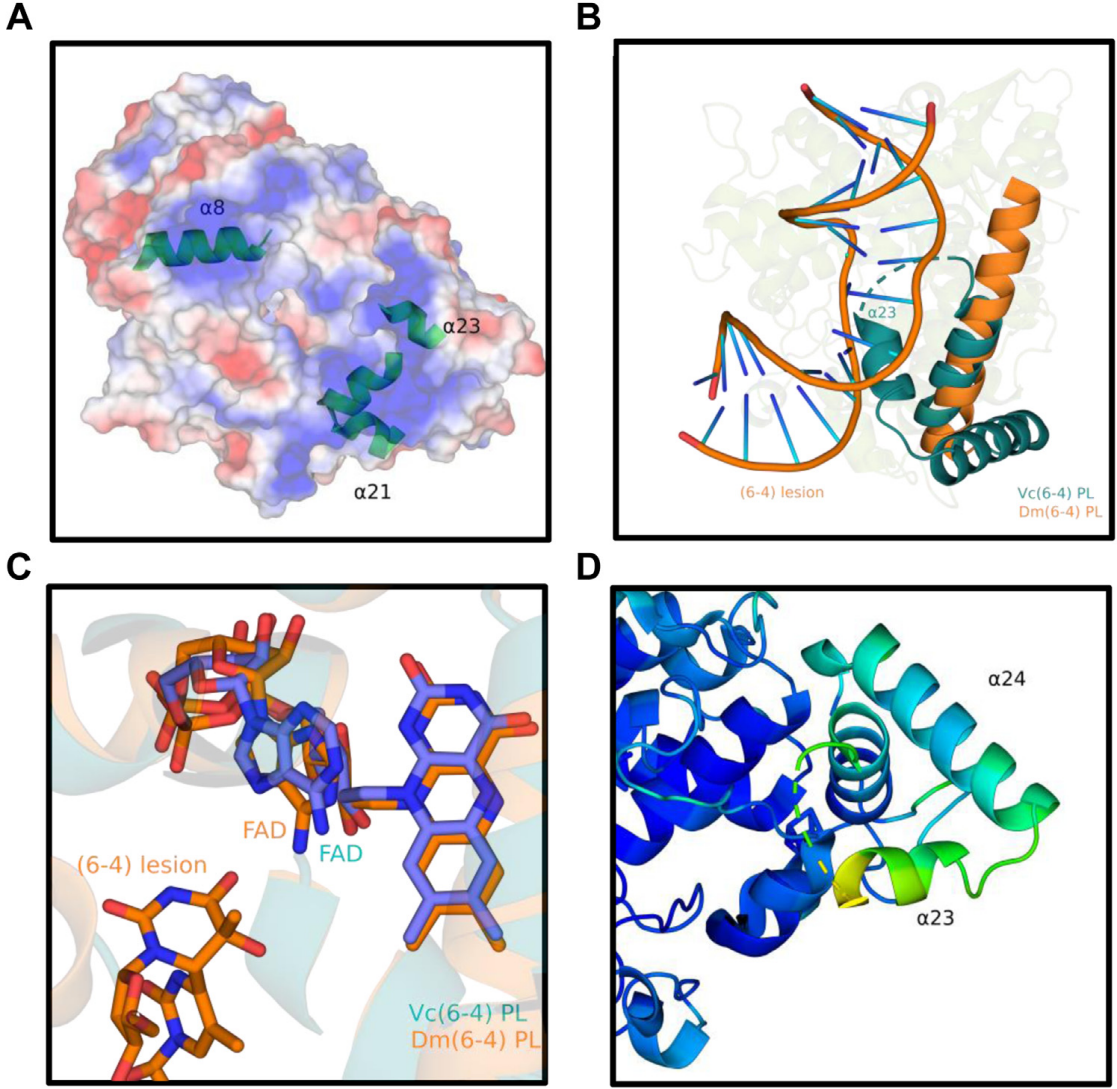
**DNA-binding comparison between *Vc* (6-4) PL and *Dm* (6-4) PL.***A*, generated surface density charge of *Vc* (6-4) PL, where *blue* is positive while *red* is negatively charged. Helices with positive surface charge are indicated in the structure. *B*, superimposed (RMSD: 3.944 Å) image of *Vc* (6-4) PL and *Dm* (6-4) PL with (6-4) DNA lesion (PDB ID: 3CVU). *Deep teal* is for *Vc* (6-4) PL, while *tv_orange* is for *Dm* (6-4) PL. *C*, FAD comparison with superimposed images of *Vc* (6-4) PL and *Dm* (6-4) PL. All atoms RMSD between the structures for FAD cofactor is 0.7 Å. *D*, B-factor analysis of the C-terminal of *Vc* (6-4) PL, where the highest B-factor has a *yellowish color*, while the lowest B-factor has a *darker blue color*. α23 has the highest B-factor in the structure. FAD, flavin adenine dinucleotide; PDB, Protein Data Bank; PL, photolyase.

Notably, we have not observed a distinct electron density of amino acid residues between 478 and 485 *Vc* (6-4) PL (Fig. 6*A*). However, this region has well-defined structures in other bacterial PLs (Fig. 6, *B–D*). Interestingly, this region is adjacent to the 23rd helix, which is one of the positively surface-charged helices around the catalytic region (Fig. 7*A*). When we superimposed *Vc* (6-4) PL with *Dm* (6-4) PL (PDB ID: 3CVU) with (6-4) T-T, this region and the 23rd helix were seen to pass through damaged DNA (Fig. 7*B*). This observation suggests that the region between 478 and 485 *Vc* (6-4) PL might be a DNA-binding region and further stabilizes after interacting with the DNA lesion. Upon investigation of the C-terminus of *Vc* (6-4) PL and *Dm* (6-4) PL, two additional helices were identified on *Vc* (6-4) PL and the 23rd helix seems to be blocking the DNA lesion in superimposed structure. Further analysis of superimposed structures indicated that the catalytic cofactor FADs are in similar conformation and their RMSD is 0.7 Å (Fig. 7*C*). To DNA lesions to bind the bacterial (6-4) PL, while still interacting with the FAD, there may be conformational changes on the 23rd helix to provide a gap for DNA lesion. B-factor analysis showed that 23rd helix has the highest B-factor values in the structure and therefore, it has the highest probability to undergo a conformational change (Fig. 7*D*). According to the superimposition of DNA lesion from *Dm* (6-4) PL with *Vc* (6-4) PL, which is compatible with a prototypical PL–DNA-binding mode, helix α23 of *Vc* (6-4) PL interacts with the DNA, while helix α24 looks away from the DNA lesion. The surface charge of helix α23 is positive, which is consistent with its role in DNA binding. The last two helixes (α23 and α24) are missing in *Dm* (6-4) PL or most of the other PLs, which indicates they may play a regulatory role rather than their involvement in repair activity.

## Discussion

PLs belong to a large family of CRY/PL responsible for repairing the UV-induced DNA damages. Different types of PLs have been discovered, and they are shown to repair two different types of DNA damages: CPDs (Pyr<>Pyr) and Pyr-Pyr photoproducts (Pyr[6–4]Pyr). CPD photoproducts are mainly repaired by CPD PLs, while (6-4) photoproducts are being repaired by (6-4) PLs (12). The reaction mechanism of CPD PLs is elucidated at *in vivo* levels (1, 5, & 6). On the other hand, different reaction mechanisms are proposed for the (6-4) PL. The (6-4) PLs are initially thought to be specifically present in eukaryotes (13). However, studies revealed that bacteria also possess (6-4) PLs (15, 16, 31). Comparison of the crystal structure of the *Vc* (6-4) PL with other known bacterial (6-4) PLs indicated that cofactor-binding regions are well conserved with some differences: a water molecule interacts with the Asp106 in *Vc* (6-4) PL with a very short distance compared to the *At* and *Rs* (6-4) PLs, where the water molecule interacts with Gly105 (Fig. 2*B*). The FAD-binding region of the *Vc* (6-4) PL is highly conserved. However, one notable difference is that Glu410 makes a hydrogen bond with Asp395; whereas, in the case of Glu399 (corresponds to Glu410 of *Vc* (6-4) PL), it makes a salt bridge with His384 (corresponds to Asp395 of *Vc* (6-4) PL) *At* (6-4) PL and *Rs* (6-4) PL (13, 16). Whether such structural difference affects its activity or conformation of FAD needs to be further investigated. The His-His-X-X-Arg motif in (6-4) PLs are shown to be important for their catalytic activity (16). This motif is also conserved in the *Vc* (6-4) PL. Further conformational comparison of the *Vc* (6-4) PL and *Dm* (6-4) PL of this motif indicates His373 and Arg376 of *Dm* (6-4) PL have identical configuration, while His372 (His364 of *Dm* (6-4) PL) acquired a different configuration, which may reveal similar/different functionalities between species.

One of the major differences between *Vc* (6-4) PL and *At* (6-4) PL structures is their predicted DNA-binding site (Fig. 5). The predicted site (amino acid residues between 178 and 185) of *Vc* (6-4) PL has a distinct structure compared to that of *At* (6-4) PL, where the corresponding region has no well-defined structure (17). The same region of *Rs* (6-4) PL also has a well-ordered electron density map except for its Arg residue (16), which indicates species-specific stability differences of DNA-binding domains. Comparison of *Vc* (6-4) PL and *At* (6-4) PL revealed that *Vc* (6-4) PL has an unstructured region with eight amino acid residues (Fig. 6*A*), which might be a DNA-binding region and is also conserved in other bacterial PLs. Arg483 might form a salt bridge with (6-4) photoproduct that stabilizes the disordered region. The general structure of *Vc* and *Dm* (6-4) PLs are highly similar except in C terminus, where *Vc* (6-4) PLs contain additional two helixes. Due to the position of the 23rd helix, DNA lesions cannot bind the *Vc* (6-4) PL similar to *Dm* (6-4) PL (Fig. 7*B*). However, the FADs are located in an extremely similar manner, which indicates a similar DNA lesion binding between the PLs (Fig. 7*C*). A conformational change upon DNA binding at the *Vc* (6-4) PL might open a space for DNA to bind. The 23rd helix is the main candidate for the conformational change, as it is the major blocker of the DNA and has the largest B-factor (Fig. 7*D*).

## Experimental procedures

### Expression and purification

Full-length (6-4) PL from *V. cholerae* O1 bivar Tor str. N16961 in a pET28a(+) vector between NdeI and BamHI cut sites was purchased from GenScript Biotech Corporation as codon optimized for *E. coli* protein expression. The plasmid was transformed into *E. coli* BL21 Rosetta-2 strain and grown in 4.5 L of LB-Miller liquid growth media supplemented with 50 μg/ml kanamycin and 35 μg/ml chloramphenicol antibiotics at 37 °C with 110 rpm shaking. When *A*600 reached to 0.8, induction of bacterial protein expression was performed by adding 0.4 mM of IPTG as the final concentration, and induction was performed at 18 °C for 24 h. The culture was centrifuged at 2850 g for 45 min at 4 °C, and the pellet was stored at −80 °C until further use. Lysis buffer containing 500 mM NaCl, 50 mM Tris–HCl pH 7.5, 10% glycerol (v/v), and 0.1% Triton X-100 (v/v) was added into pellets, and sonication (Branson W250 sonifier) was performed for 45 s at 60% power 3 times. Samples were ultracentrifuged at 35,000 rpm with a Ti-45 rotor (Beckman Coulter) for 1 h at 4 °C; subsequently, the supernatant was filtered with a 45 μm cellulose mixed ester filter (ISOLAB) and then applied to Ni-NTA agarose resin (QIAGEN). The column was equilibrated with His A buffer containing 200 mM NaCl, 20 mM Tris–HCl pH 7.5 (v/v), and 5% glycerol (v/v); then the sample was loaded with 2.5 ml/min flow rate. Washing of the column was performed with His A buffer, and protein was eluted with His B buffer, containing 200 mM NaCl, 20 mM Tris–HCl pH 7.5, 250 mM imidazole, and 5% glycerol (v/v) into 5 ml of 100% glycerol to prevent protein precipitation. Yellow-colored (6-4) PL was flash frozen with liquid nitrogen and stored at −80 °C with a final concentration of 25% glycerol (v/v).

### Crystallization and harvesting

Initial crystallization screening experiments were setup with a 5 mg/ml final concentration of *Vc* (6-4) PL with the sitting drop, microbatch screening (under oil) method at 4 °C by mixing 0.83 μl of protein with an equal volume of commercial crystallization screening conditions in 72-well Terasaki plates. The well containing the protein:cocktail mixture was covered with 16.6 μl of parafilm oil to allow slow evaporation of solvents. Approximately, 3500 commercially available crystal screening conditions were tested, and the yellow-colored crystals were observed at Wizard Synergy #40 (Rigaku Corporation), which contains 2 M of ammonium citrate/citric acid pH 7.5 and 5% PEG 400 (v/v) after 7 weeks. The conditions were further optimized by mixing 0.5 μl Cryo-Pro #45 (Hampton Research), which contain 1 M sodium sulfate decahydrate, 1 μl of protein, and 1 μl of Wizard Synergy #40. The crystals were flash frozen by quickly plunging them into liquid nitrogen, and data collection was performed at 100 K.

### Data collection and processing

Two X-ray diffraction datasets were collected at 2.0 Å and 2.5 Å resolutions from two large crystals with a wavelength of 1.54 Å at the University of Health Sciences with Rigaku’s XtaLAB Synergy Flow X-ray diffractometer (Rigaku Corporation). The PhotonJet-R (Rigaku Corporation) X-ray generator was operated at 40 kV and 30 mA with a 23% beam intensity to mitigate the radiation damage during data collection. HyPix-Arc-150° detector (Rigaku Corporation) was used with a 60 mm detector distance. To further minimize the exposure time, we preferred not to collect fine-sliced oscillation data rather 1-degree oscillation scan width low X-ray dose chosen with 15 and 30 s exposure times. The total run time for two data collections was 1 h 11 m 30 s and 2 h 33 min 0 s, respectively. Profit merge is done by using the data from both crystals and data reduction is performed with CrysAlis Pro (32) software version 171.42.51a (https://www.rigaku.com/products/crystallography/crysalis). Two datasets were merged with 99.6 % completeness and 60.6 fold multiplicity. Unit cell dimensions were a = 200.8 Å, b = 200.8 Å, c = 77.0 Å, α = 90, β = 90, and γ = 120, in space group P6_4_ 22.

### Structure determination and refinement

Swiss model (33) was used for building a search model for structure determination that gave the best result with PDB ID: 4DJA (13), which was used in the automated molecular replacement program *PHASER* (34) in *PHENIX* (35) software version 1.20.1-4487 (https://phenix-online.org/). Further refinements were performed with *PHENIX*, and the addition of water and cofactors was performed by using *COOT* (36) software version 0.9.6 (https://www2.mrc-lmb.cam.ac.uk/personal/pemsley/coot/). The final R_work_ is 22.54% and R_free_ is 28.30%, completeness is 99.60% with 24.250 to 2.500 Å refinement resolution (Table 1). The structure contains no Ramachandran outliers with 98.01% of the residues in the favored regions. The structure was deposited to the RCSB PDB website with the PDB ID: 7YKN. Figures were generated at *PyMOL* software (DeLano Scientific) version 2.4.1 (https://pymol.org/2/). Data collection and structure determination information are summarized in Table 1.

**Table 1:** Statistics for data collection, processing, and structure refinement

Beamline	Turkish light source (Turkish DeLight)
Resolution range	24.25-2.5 (2.589–2.5)
Space group	P6_4_ 2 2
Unit cell	200.8200.8 77.0 90 90,120
Total reflections	1,935,882 (189,833)
Unique reflections	32,043 (3130)
Multiplicity	60.4 (60.6)
Completeness (%)	99.60 (99.97)
Mean I/σ	5.61 (1.70)
Wilson B-factor	15.97
R-merge	1.382 (3.765)
R-meas	1.394 (3.796)
R-pim	0.1776 (0.4855)
CC 1/2	0.955 (0.546)
CC^a^	0.988 (0.84)
Reflections used in refinement	31,969 (3130)
Reflection used for R-free	1068 (105)
R-work	0.2254 (0.2891)
R-free	0.2830 (0.3620)
CC (work)	0.910 (0.712)
CC (free)	0.929 (0.542)
Number of non-hydrogen atoms	4778
Macromolecules	4120
Ligands	101
Solvent	557
Protein residues	507
RMS (bonds)	0.002
RMS (angles)	0.48
Ramachandran favored (%)	98.01
Ramachandran allowed (%)	1.99
Ramachandran outliers (%)	0.00
Rotamer outliers (%)	1.84
Clashscore	5.92
Average B-factor	22.46
macromolecules	22.31
Ligands	17.61
Solvent	24.49
Number of TLS groups	8

a Pearson correlation coefficient (CC).

## Data availability

All data generated or analyzed during this study are included in this article, and the structure was deposited to the RCSB PDB website with the PDB ID: 7YKN.

## Conflict of interest

The authors declare that they have no conflicts of interest with the contents of this article.
